# User-Reported Mechanisms of Change on a Suicide Prevention Website: Single-Arm Pragmatic Trial

**DOI:** 10.2196/71136

**Published:** 2025-09-25

**Authors:** Martina Fruhbauerova, David Huh, Ursula Whiteside

**Affiliations:** 1 Department of Psychology University of Kentucky Lexington, KY United States; 2 Now Matters Now Bellevue, WA United States; 3 Department of Psychiatry and Behavioral Sciences School of Medicine University of Washington Seattle, WA United States

**Keywords:** suicide, dialectical behavior therapy, digital mental health, web-based interventions, suicide prevention, digital accessibility internet, help-seeking behavior, behavior therapy, crisis intervention

## Abstract

**Background:**

Digital platforms can serve as effective interventions for individuals in crisis, those with limited access to mental health resources, or those who prefer web-based support over in-person care. NowMattersNow.org, a web-based platform grounded in dialectical behavior therapy, has been shown to reduce suicidal thoughts and negative emotions. However, little is known about the specific mechanisms that drive these improvements. Identifying the active ingredients that contribute to its effectiveness will help optimize its impact.

**Objective:**

This study examined the reasons users reported behind reductions in suicidal thoughts and negative emotions after visiting NowMattersNow.org. Specifically, this study sought to determine which reported reasons were associated with greater versus lesser improvements and whether these changes differed across specific subgroups.

**Methods:**

In this single-arm pragmatic trial, data were collected from 3185 respondents who completed a 6-item retrospective survey while visiting NowMattersNow.org. The survey assessed changes in suicidal ideation and emotional distress (ie, intensity upon entering the site vs at the time of survey completion), reasons the website was helpful, and basic nonexclusive demographic information. Cross-tabulations were used to examine the most commonly endorsed reasons for finding the website helpful, while longitudinal regression analyses assessed the statistical significance of changes in suicidal ideation and emotional distress.

**Results:**

The majority of participants reported experiencing suicidal thoughts (n=2309, 72.5%) and negative emotions (n=2745, 86.2%) upon arriving at the website, with 52.4% (n=1211) and 55.6% (n=1527) of these individuals, respectively, experiencing reductions in suicidal thoughts and negative emotions after engaging with the site. Regarding the primary aims of the study, the most frequently cited reason for finding NowMattersNow.org helpful was “I learned something” (n=668, 21%), followed by “It distracted me” (n=544, 17.1%) and “I felt less alone” (n=414, 13%). These were also the top 3 reasons reported by LGBTQI individuals, those endorsing alcohol or opioid problems, and those experiencing unusual experiences, though the order varied across groups. Among participants who experienced the largest reduction in suicidal ideation (a 4-point decrease), the most common reasons cited were “It distracted me” (n=5, 29.4%), “I felt less alone” (n=3, 17.6%), and “I felt cared for” (n=3, 17.6%). Similarly, for those with the largest reduction in negative emotions (a 4-point decrease), the most frequently endorsed reasons were “It distracted me” (n=3, 23.1%), “I felt less alone” (n=3, 23.1%), and “I felt cared for” (n=2, 15.4%).

**Conclusions:**

The findings suggest that NowMattersNow.org is an accessible, scalable digital intervention that shows promise for reducing suicidal ideation and emotional distress, particularly in vulnerable populations. Key elements, such as fostering social connectedness, distraction, and educational content, appear to be critical components of its effectiveness, indicating that web-based self-help tools like NowMattersNow.org can provide short-term management of suicidal thoughts and negative emotions.

## Introduction

### Background

Suicide is a leading cause of death in the United States and represents a major public health crisis [1]. In 2022, over 13.2 million individuals in the United States seriously thought about suicide, 1.6 million attempted suicide, and 49,000 people died by suicide [2]. Many of these individuals had seen a health care provider in the month prior to their death [3,4]. However, many providers may not have the opportunity to intervene due to several factors: many individuals are uncomfortable discussing their suicidal thoughts with providers [5,6], suicidal ideation can fluctuate rapidly [7,8], and some suicide attempts are entirely unplanned [9-11]. Importantly, people experiencing suicidal thoughts often turn to the internet for support and information, with use tending to rise as suicidal thoughts intensify [12-15]. These findings reinforce the need for readily accessible and effective digital resources for those reaching out during critical moments. Indeed, researchers are calling for suicide-specific web-based tools for short-term management of suicidal thoughts and negative emotions [16].

Despite significant progress in developing empirically supported psychological treatments, a large proportion of people with mental health issues still does not receive these interventions. Closing this treatment gap is crucial. Web-based resources present scalable and accessible solutions that have the potential to extend help to a broader population and improve access to necessary mental health support. For example, a meta-analysis of 66 randomized controlled trials examining app-supported smartphone interventions for mental health problems found these platforms to be highly effective [17]. Specifically, smartphone interventions significantly outperformed control conditions in reducing depressive symptoms, generalized anxiety, stress, social anxiety, and general psychiatric distress, while also improving quality of life and positive affect. Importantly, these smartphone-based interventions were similar in their effectiveness to traditional active treatments, such as face-to-face or computerized therapy.

Several systematic reviews and meta-analyses have shown promising results for web-based suicide-specific prevention strategies as well. One systematic review and meta-analysis found that self-guided digital interventions aimed directly at reducing suicidal ideation were effective immediately after the intervention [18]. Another review indicated that, although there was not yet enough evidence to classify web-based cognitive behavioral therapy (CBT), mindfulness-based CBT, and dialectical behavioral therapy as evidence-based treatments for suicidal ideation and self-harm, digital interventions could be a safe and acceptable alternative for those unable or unwilling to engage in face-to-face therapy [19]. A separate systematic review and meta-analysis using individual participant data supported the efficacy of digital CBT in reducing suicidal ideation across different ages, genders, and histories of suicide attempts, though data on its effects on actual suicide attempts were limited [20]. Another review of web-based and mobile apps designed to prevent suicidal ideation and self-harm showed that digital interventions were linked to reductions in suicidal ideation scores postintervention [21]. However, one meta-analysis suggested that while internet- and mobile-based interventions had a positive effect on reducing suicidal ideation compared to usual treatment, this effect was not significant when compared to active control groups [22].

Similarly, a systematic review of mobile apps (such as iBobbly, Virtual Hope Box, BlueIce, and Therapeutic Evaluative Conditioning) found positive outcomes for individuals at high risk for suicide or self-harm, including reduced depression, psychological distress, and self-harm, and improved coping self-efficacy [23]. However, none of these apps significantly reduced suicidal ideation compared to a control condition. Finally, another systematic review of mobile- and internet-based interventions for self-harm showed that these interventions were generally well-accepted and associated with reductions in suicidal thoughts and behaviors (STBs) compared to treatment as usual [22]. However, their efficacy was not significant when compared to trials with active control groups. In summary, these systematic reviews and meta-analyses suggest that digital interventions can be a safe, acceptable, and potentially effective option for reducing STBs, particularly for individuals who may not access traditional in-person treatments. However, their effectiveness compared to active control conditions remains mixed, highlighting the need for further research to better understand the contexts in which these digital approaches are most effective.

Dialectical behavior therapy (DBT) is a well-established, evidence-based treatment for suicidal behaviors [24], making it a promising candidate for adaptation into scalable and accessible web-based interventions. Originally developed for individuals with borderline personality disorder who experience chronic suicidal thoughts and who engage in nonsuicidal and suicidal self-injury [25], DBT is grounded in the biosocial theory [25]. This theory posits that borderline personality disorder arises from the interaction between biologically predetermined emotional vulnerability (characterized by heightened emotional reactivity, intensity, and slow return to baseline) and invalidating environments, which can range from inattentive caregivers to physical or sexual abuse. This interplay impairs the individual’s ability to trust, understand, and regulate emotions, leading to maladaptive behaviors that serve as escape mechanisms—suicidal behavior being the most extreme form.

Given DBT’s strong emphasis on targeting STBs, digital adaptations hold significant potential for expanding access to evidence-based suicide interventions. However, there is limited evidence supporting its effectiveness for managing STBs through internet-based platforms. Still, some digital tools incorporating DBT principles have already demonstrated positive outcomes. For instance, CALMA, a DBT-based app designed to reduce nonsuicidal and suicidal self-directed violence, has exhibited reductions in suicidal ideation, suicide plans, suicidal gestures, thoughts about nonsuicidal self-injury, and nonsuicidal self-injury itself from pre- to postintervention [26]. Similarly, an internet-delivered DBT skills training program for suicidal individuals with heavy episodic drinking showed significant reductions in suicidal ideation, alcohol use, and emotion dysregulation over 4 months [27]. Additionally, DBT Coach, a mobile app aimed at improving skills generalization through digital DBT skills coaching, was found to reduce subjective distress and urges to self-harm following app use [28]. Furthermore, other studies have indicated that delivering DBT through web-based platforms or apps is feasible, accessible, acceptable, and safe [29], and is generally well-received by participants [30]. In contrast, a pragmatic randomized clinical trial challenged the effectiveness of web-based DBT skills training [31]. Among adult outpatients with frequent suicidal ideation, those who received an invitation to skills training had approximately 30% higher risk of self-harm compared to those receiving usual care. This increased risk may be attributed to the trial’s pragmatic design, where only 30% to 40% of participants actively engaged with the offered interventions. Among those who did engage, “active” participation was minimal; fewer than 10% viewed content beyond the brief introductory video, and only 2% sustained engagement for more than 9 months. These findings suggest that the unfavorable results may stem from underutilization of the service rather than the intervention’s ineffectiveness, highlighting the importance of addressing barriers to sustained engagement in web-based treatments. Although the evidence for internet-based DBT interventions for STBs is still limited and mixed, several digital tools incorporating DBT principles have shown promising outcomes in reducing suicidal ideation, self-harm, and related symptoms, highlighting their potential for bridging the gap in immediate assistance for individuals at risk for suicide.

In addition to digital DBT interventions, there has been a rise in self-help websites aimed at making DBT resources more widely available, such as dbtselfhelp.com, dialecticalbehaviortherapy.com, dbt.tools, or dbtbites.com. While these websites hold significant potential for expanding access to web-based DBT resources, they lack empirical evidence supporting their effectiveness [32]. One exception is NowMattersNow.org, which was designed to support individuals experiencing STBs, as well as their providers. This website is grounded in DBT principles and features personal stories of successful coping and recovery from suicidal thoughts and painful emotions. In their study evaluating the utility of the website [16], 70% of survey respondents reported suicidal ideation upon entering the website. The results indicated a statistically significant reduction in suicidal thoughts, with an average decrease of 0.21 points (*d*=0.13) on a 1 to 5 scale, and a reduction in negative emotions of 0.32 points (*d*=0.21). Importantly, individuals with high suicidal ideation (ratings of 4 or 5) experienced a reduction of 6.4% points (*d*=0.13), while those with high negative emotions saw a 10.9% point reduction (*d*=0.23). Despite the small effect sizes, these reductions are still clinically significant, as even modest improvements in suicidal ideation and negative emotions, along with access to crisis management tools, could potentially impact individuals’ ability to manage their distress and prevent suicidal behaviors.

Given the promising outcomes associated with NowMattersNow.org and other digital interventions aimed at suicide prevention, it raises important questions about the active ingredients and content features that drive their effectiveness. Understanding the mechanisms behind the reductions in suicidal ideation and negative emotions observed on NowMattersNow.org is crucial for optimizing its impact. Some potential mechanisms have been proposed in the suicidology field that may explain the effectiveness of such interventions. For example, “thwarted belongingness,” or the sense of disconnection from others, is a well-recognized risk factor for suicidal ideation in major suicide theories [33,34]. This construct also aligns with the biosocial theory [25], in which chronically invalidating environments contribute to interpersonal difficulties, leaving individuals feeling isolated and disconnected. The videos on NowMattersNow.org, which feature individuals sharing their personal journeys through suicidal ideation and recovery, may counteract this disconnection, fostering a sense of connection and reducing feelings of isolation. Additionally, the putative mechanism of “caring contacts [35]”—the only intervention shown to have reduced suicide deaths in clinical trials [36,37]—is thought to operate through strengthening social connection [38]. In this way, NowMattersNow.org may help visitors feel less alone, which could partially explain the observed reductions in suicidal ideation and negative emotions. However, further investigation is needed to identify which features of the website contribute most effectively to these outcomes. Finally, it is important to examine whether high-risk and marginalized populations, who are often underrepresented in digital mental health research, benefit from interventions like NowMattersNow.org. If effective, such platforms could serve as a critical resource for reaching individuals who might otherwise have limited access to evidence-based suicide prevention support.

### This Study

The next step in evaluating the effectiveness of the NowMattersNow.org website is to determine what features of the website are the most useful and effective in order to expand and refine the content so that the website’s effectiveness can be improved even further, potentially saving lives. Additionally, discerning what users find helpful would also provide opportunities for future content personalization. For example, if some individuals benefit most from feeling less isolated or are encouraged to call a crisis line, the website could be tailored in the future to meet these specific needs. As such, this study was designed to extend the previous work of Whiteside et al [16] and determine the reasons for reductions in suicidal thoughts and negative emotions after visiting the NowMattersNow.org website. We hypothesized that participants who reported decreased suicidal thoughts and negative emotions after using the website would primarily attribute their improvement to feeling “less alone” from the content provided. We also aimed to investigate which reported reasons were associated with a greater or lesser magnitude of improvements in suicidal ideation and negative emotions. Due to the limited literature on the mechanisms through which websites make an impact, we did not have a specific hypothesis regarding which reasons would be associated with varying degrees of improvement. Finally, we investigated whether the changes in suicidal ideation and negative emotions differed across specific subgroups. As this was an exploratory aim, we did not generate a priori hypotheses for these analyses.

## Methods

### Study Design

This study used a single-arm pragmatic trial design, leveraging quality improvement data from the NowMattersNow.org web-based platform. The website and survey were not originally created for research purposes, nor were they designed for any specific study. Participants were real-world users of the site, with natural variations in how they engaged with the content. This approach allowed for a more ecological and generalizable assessment of the website’s impact, reflecting typical user interactions.

### Procedures

Participants for this study were recruited through the NowMattersNow.org website. The survey was designed to activate after a specific duration of engagement with the site: 1 minute on mobile devices, and initially 3 minutes on desktop and tablet devices, which corresponds to the average length of one short video. However, due to limitations in the technology at the time, the survey could only be set to trigger after 1 minute of engagement on mobile devices. Unfortunately, this created discrepancies in the activation times between mobile and desktop users. In 2015, the activation time for desktop and tablet users was extended to 8 minutes—approximately the length of 2 short videos or 1 longer video—to encourage deeper engagement with the content before prompting the survey. While we could confirm that participants remained on a page long enough to trigger the survey, we could not track their total time spent across multiple pages due to the absence of cookies; this limitation was implemented to protect user privacy, given the sensitive nature of the website’s content. Once the survey was activated, users were required to complete it before proceeding to other site features, although they could close the survey window using a button located in the upper right corner. After a survey was displayed—regardless of whether it was completed or not—participants would not be prompted again for at least 24 hours. We did not track how often the survey appeared without being completed. Prior to the launch of the study and data collection, the survey was rigorously tested by developers at Civilization [39] to ensure functionality and usability. Throughout this process, our only interaction with participants was through their survey responses, with no direct contact before or after submission.

### NowMattersNow.org

The website aims to equip individuals experiencing suicidal thoughts and health care providers with DBT skills, serving as a valuable resource for supporting suicidal patients. The long-term objective is for providers to use these skills in their own lives and teach them to patients, thereby helping to prevent and manage suicidal crises. Consequently, the website was developed with health care providers in mind as a tool to improve care for suicidal patients. To illustrate how individuals can receive support, visitors might search for DBT skills and find real-life examples of how others have successfully used these skills to cope with distressing emotions, offering both guidance and validation. Additionally, the site provides access to a variety of crisis helplines tailored to different groups, helping users connect to appropriate support based on their needs. Many users also report feeling comforted by hearing personal recovery stories shared by others who have faced similar challenges. The NowMattersNow.org landing page, visible during the study period, reassures visitors with the message, “Have you had suicidal thoughts? Problems that felt unsolvable? You are in excellent company—we’ve been there.” Further details on the origins and development of the NowMattersNow.org website can be found in the original study [16].

### Measures

NowMattersNow.org routinely collects survey data during user sessions to improve quality and assess the website’s impact on various populations. The survey consists of 7 items developed using HTML elements within WordPress and triggered by JavaScript. It is not publicly advertised and does not require a user login, ensuring confidentiality. The survey begins with a header stating, “We’d appreciate your confidential feedback to improve our site.”

Users are prompted to retrospectively evaluate their “Intensity of negative emotions” and “Intensity of suicidal thoughts” on a 5-point scale from 1 to 5, ranging from “Not at all” to “Completely overwhelming.” The survey captures both baseline and postwebsite ratings within a single session, asking participants to report their emotional state at 2 time points: “When you entered this site” (baseline) and “What level are they now?” (postwebsite use). As such, participants were asked to retrospectively report their emotional state before arriving at the website and then rate their current state after engaging with the site. Since the survey is completed in one sitting, participants who do not finish it would have incomplete responses. Additionally, users can retake the survey after 24 hours. These survey items were adapted from DBT skills “diary cards,” which patients use to track suicidal thoughts, mood, and related behaviors.

Following these assessments, users are asked to identify what aspects of NowMattersNow.org they found helpful, selecting the top reason for feeling at least a little better. The options provided include: “It distracted me,” “I felt less alone,” “I learned something,” “I called/texted a crisis line,” “I saw that others could recover,” “I felt cared for,” “Time passed,” “It wasn’t helpful,” and “Other.” No follow-up question was included to further explore the “Other” responses provided by participants.

The survey also collects basic demographic information using nonexclusive checkboxes to identify users who identify as native or Indigenous, LGBTQI, or who have experienced alcohol or opioid problems, or unusual experiences. The term “unusual experiences” was not further defined for participants, but it was intended to capture a broad range of psychological phenomena (eg, hallucinations and mania) in everyday language, avoiding the use of diagnostic terminology.

Although survey responses were not assessed for completeness prior to submission (ie, incomplete responses were allowed), only participants who provided complete responses on their suicidal thoughts and negative emotions were included in the analysis (ie, if users did not select the top reason for feeling at least a little better, and they were still included in the analyses and their reason was coded as “No response”). Once users clicked the “submit” button, their responses became final. Further details about the study measures can be found in the original paper [16].

### Ethical Considerations

This study was approved by the Allendale Institutional Review Board (Old Lyme, Connecticut; IRB #: 20180223W1, Title: “Review of NowMattersNow.org Survey Data”). This study used previously collected quality improvement data from the NowMattersNow.org website. As the data were recorded in a manner that ensured participants could not be identified, either directly or through linked identifiers, the study qualified for an exemption from the HHS human participant regulations (under 45 CFR 46.101(b)(4)). Since no protected health information was collected as part of the quality improvement process, the study met the criteria for exemption, and informed consent was not required. Participants were given the option to skip questions or close the window if they did not wish to participate, ensuring their ability to opt out. To maintain privacy and confidentiality, IP address snippets were removed, and each participant was assigned a unique identification number. The final deidentified dataset was stored securely on a password-protected Google Drive. No compensation was provided to participants for taking the survey on the NowMattersNow.org platform.

### Participants

This study used a convenience sample derived from data from 5539 survey responders who visited NowMattersNow.org, collected from May 14, 2020, to September 12, 2023. During this period, there were no major modifications to the website; only minor updates, such as the addition of short videos, may have occurred. However, because web designers and content developers did not systematically track these changes, we do not have records detailing what modifications were made or when they took place. Overall, it is reasonable to assume that all participants were exposed to the same fundamental content throughout the study period. Additionally, we did not track how long survey respondents remained on the website beyond completing the survey.

In cases where multiple survey responses originated from the same IP address, only the first response was retained to ensure that the sample represented unique individuals. Due to this approach, some individuals may have been assigned different IP addresses if they accessed the website from different locations, while others may have shared the same IP address if they were using a communal network (eg, within the same facility). We focused our analysis on the subset of 3374 survey respondents who did not identify as a mental health care provider. Our final analytic sample included 3185 survey respondents who answered 4 questions regarding suicidal ideation and negative emotions (baseline and postwebsite). The final dataset was stored in Google Drive, to which authors MF, DH, and UW had access.

### Analytic Approach

First, preliminary analyses were conducted to characterize the degree of missing data and whether participant characteristics differed when outcome data were complete versus incomplete. These descriptive analyses informed how to address missing data in subsequent inferential analyses. Second, longitudinal regression analyses using generalized estimating equations evaluated whether the magnitude and statistical significance of user-reported changes in suicidal ideation and negative emotion varied for specific subgroups, including those who (1) identified as Native or Indigenous, (2) identified as LGBTQI, (3) endorsed alcohol or opioid misuse, and (4) endorsed unusual experiences (eg, psychotic symptoms). An exchangeable working correlation matrix in conjunction with cluster robust standard errors accounted for the correlation of repeated measures (baseline and post) within participants.

The following equation represents the model for the suicidal ideation outcome:

SuicidalIdeation_ti_ = *b*_0_ + *b*_1_Indigenous_ti_ + *b*_2_LGBTQI_ti_ + *b*_3_AlcoholOpioidMisuse_ti_ + *b*_4_UnusualExperiences_ti_ + *b*_5_Time_ti_ + *b*_6_(Indigenous_ti_ × Time_ti_) + *b*_7_(LGBTQI_ti_ × Time_ti_) + *b*_8_(AlcoholOpioidMisuse_ti_ × Time_ti_) + *b*_9_(UnusualExperiences_ti_ × Time_ti_),

Where *t* indexes time point and *i* indexes individual. The predictors were coded as follows: time (0=baseline, 1=postwebsite), Indigenous identity (0=no, 1=yes), LGBTQI identity (0=no, 1=yes), alcohol or opioid misuse (0=no, 1=yes), and unusual experiences (0=no, 1=yes). This parameterization evaluated all of the subgroup effects in a single regression model and accommodated participants who belonged to multiple subgroups. The equation for the negative emotions outcome was parameterized similarly. Thus, all of the subgroup characteristics assessed in the survey (ie, Indigenous identity, LGBTQI identity, alcohol or opioid misuse, and unusual experiences) were included as covariates in each outcome analysis.

Third, we evaluated cross-tabulations of user-reported reasons why NowMattersNow.org was helpful by (1) the baseline severity of suicidal ideation or negative emotions (1=no symptoms, 2 to 3=moderate symptoms, and 4 to 5=high symptoms). This categorization was based on a core tenet of DBT—the diary card [25]. While the DBT manual does not explicitly define these categories, we drew on the manual’s guidance, as well as our clinical judgment (2 authors are clinicians) and lived experience, to define these symptom severity ranges. Additionally, (2) we examined the change in suicidal ideation or negative emotions from baseline to postwebsite (improved, did not change, or worsened).

Statistical significance was evaluated based on the conventional threshold of *P*<.05.

## Results

### Preliminary Analyses

Of the 3374 eligible survey responses from nonproviders, 3185 (94.4%) had complete data on suicidal ideation and negative emotions (baseline and postwebsite), and 189 (5.6%) had incomplete outcome data. Participants with complete outcome data were more likely to identify as LGBTQI (n=1034, 32.5% vs n=24, 12.7%; *χ*^2^_1_=32.4; *P*<.001) and more likely to endorse having experienced unusual experiences (n=1054, 33.1% vs n=36, 19%; *χ*^2^_1_=16.1; *P*<.001) than those with incomplete outcome data. There was no statistically significant difference between respondents with complete versus incomplete outcome data with respect to indigenous identity (n=147, 4.6% vs n=10, 5.3%; *χ*^2^_1_=0.2; *P*=.67) or endorsement of alcohol or opioid problems (n=286, 9% vs n=10, 5.3%; *χ*^2^_1_=3.0; *P*=.08).

The outcome analyses focused on the 3185 responses with complete outcome data, as this increased the representation of at-risk subgroups (ie, LGBTQI, and endorsement of potential psychotic symptoms). In the final analytic sample, 32.5% (n=1034) identified as LGBTQI, 5% identified as Native or Indigenous, 33.1% (n=1054) endorsed having experienced unusual experiences, and 9% (n=286) reported alcohol or opioid problems. As noted in the Analytic Approach section, the 4 participant characteristics assessed in the survey were included as covariates in the outcome analyses, so subgroup effects in the outcome analyses were adjusted for other participant characteristics.

### Descriptive Analyses: Changes in Suicidal Ideation and Negative Emotions

Table 1 summarizes the intensity of and changes in suicidal thoughts and negative emotions, as well as reasons for NowMattersNow.org being helpful within-participant subgroups and overall across all respondents (n=3185).

At the time of entry, 2309 (72.5%) participants endorsed at least some suicidal ideation, defined as a rating greater than 1 on a 5-point scale (1=not at all; 5=completely overwhelming). Similarly, 2745 (86.2%) participants reported experiencing at least some degree of negative emotions.

Following engagement with NowMattersNow.org, 1211 (38%) participants reported improvements in the intensity of suicidal thoughts; when considering only those who initially endorsed suicidal ideation (n=2309), 52.4% (n=1211) experienced a reduction. Overall, there was a 0.5-point reduction in the intensity of suicidal thoughts, from an average of 3.0 at baseline to 2.5 after viewing the website (1=not at all, 5=completely overwhelming). There was a greater reduction in suicidal ideation among (1) those who identified as LGBTQI (vs non-LGBTQI; estimate –0.21, 95% CI –0.28 to –0.14; *P*<.001), (2) those who endorsed alcohol or opioid problems (vs those without alcohol or opioid problems; estimate –0.13, 95% CI –0.24 to –0.02; *P*=.02), and (3) those who endorsed unusual experiences (vs those without unusual experiences; estimate –0.11, 95% CI –0.18 to –0.04; *P*=.001).

With respect to negative emotions, 1527 (47.9%) participants reported improvements in the intensity of negative emotions; among those who initially endorsed negative emotions, 55.6% (n=1527) experienced a reduction. Further, respondents reported a 0.62-point reduction from an average of 3.66 at baseline to 3.04 after viewing the website. There was a greater reduction in negative emotions among (1) those who identified as LGBTQI (vs non-LGBTQI; estimate –0.15, 95% CI –0.22 to –0.08; *P*<.001) and (2) those who endorsed unusual experiences (vs those without unusual experiences; estimate –0.09, 95% CI –0.17 to –0.02; *P*=.01).

**Table 1:** Descriptive summary of the intensity of and change in suicidal thoughts and negative emotions, and reasons endorsed for NowMattersNow.org being helpful: overall and by participant characteristics (n=3185)^a^.

			Native or Indigenous (n=147)		LGBTQI (n=1034)		Alcohol or opioid problem (n=286)		Unusual experiences (n=1054)		Overall (n=3185)
**Intensity of suicidal thoughts, mean (SD)**											
	Previsit	3.22 (1.58)		3.47 (1.43)		3.38 (1.41)		3.18 (1.50)		3.03 (1.54)	
	Postvisit	2.59 (1.43)		2.79 (1.40)		2.70 (1.38)		2.55 (1.41)		2.51 (1.43)	
**Change in suicidal thoughts, n (%)**											
	Improved	59 (40.1)		492 (47.6)		141 (49.3)		455 (43.2)		1211 (38)	
	No Change	85 (57.8)		514 (49.7)		132 (46.2)		566 (53.7)		1882 (59.1)	
	Worsened	3 (2)		28 (2.7)		13 (4.5)		33 (3.1)		92 (2.9)	
**Intensity of negative emotions, mean (SD)**											
	Previsit	3.86 (1.38)		3.98 (1.22)		3.97 (1.17)		3.83 (1.31)		3.66 (1.41)	
	Postvisit	3.08 (1.35)		3.24 (1.23)		3.25 (1.24)		3.13 (1.31)		3.04 (1.37)	
**Change in negative emotions, n (%)**											
	Improved	76 (51.7)		573 (55.4)		157 (54.9)		551 (52.3)		1527 (47.9)	
	No Change	65 (44.2)		417 (40.3)		102 (35.7)		443 (42)		1495 (46.9)	
	Worsened	6 (4.1)		44 (4.3)		27 (9.4)		60 (5.7)		163 (5.1)	
**Perceived helpfulness of NowMattersNow.org, n (%)**											
	Helpful (specific and nonspecific)	126 (85.7)		855 (82.7)		243 (85)		885 (84)		2555 (80.2)	
	Not helpful	10 (6.8)		82 (7.9)		19 (6.6)		443 (42)		218 (6.8)	
	No Response	11 (7.5)		97 (9.4)		24 (8.4)		97 (9.2)		412 (12.9)	
**What about NowMattersNow.org was helpful? n (%)**											
	I learned something^b^	32 (21.8)^c^		155 (15)^d^		49 (17.1)^e^		190 (18)^d^		668 (21)^c^	
	It distracted me^b^	28 (19)^e^		215 (20.8)^c^		50 (17.5)^c^		205 (19.4)^c^		544 (17.1)^e^	
	I felt less alone^b^	16 (10.9)^d^		161 (15.6)^e^		45 (15.7)^d^		151 (14.3)^e^		414 (13)^d^	
	Time passed	9 (6.1)		100 (9.7)		34 (11.9)		103 (9.8)		252 (7.9)	
	Other	9 (6.1)		56 (5.4)		21 (7.3)		67 (6.4)		238 (7.5)	
	It wasn’t helpful	10 (6.8)		82 (7.9)		19 (6.6)		72 (6.8)		218 (6.8)	
	I called/texted a crisis line	13 (8.8)		88 (8.5)		19 (6.6)		83 (7.9)		197 (6.2)	
	I felt cared for	9 (6.1)		50 (4.8)		15 (5.2)		48 (4.6)		127 (4)	
	I saw that others could recover	10 (6.8)		30 (2.9)		10 (3.5)		38 (3.6)		115 (3.6)	
	No response	11 (7.5)		97 (9.4)		24 (8.4)		97 (9.2)		412 (12.9)	

^a^The sample sizes across the 4 subgroups do not add up to the overall n=3185 since respondents could endorse a single, multiple, or no subgroups.

^b^Top 3 reasons.

^c^Most frequently endorsed reason.

^d^Third most frequently endorsed reason.

^e^Second most frequently endorsed reason.

### Main Outcome Analyses

With respect to the perceived helpfulness of NowMattersNow.org, 80.2% (n=2555) of all respondents reported that the website was helpful. Overall, the most common frequently endorsed reason why NowMattersNow.org was helpful was that “I learned something” (n=668, 21%), followed by “It distracted me” (n=544, 17.1%), and “I felt less alone (n=414, 13%). These were also the top 3 reasons across all subgroups, although “It distracted me” and “I felt less alone” were the top 1 and 2 reasons, respectively, among those identifying as LGBTQI, those that endorsed alcohol or opioid problems, and those that endorsed unusual experiences.

Tables 2 and 3 summarize the reasons for NowmattersNow.org being helpful by (1) the intensity of baseline symptoms (suicidal thoughts or negative motions) and (2) the change in symptoms (improved, no change, worsened). Overall, 38% (n=1211) of all respondents reported a reduction in suicidal thoughts, and 47.9% (n=1527) reported a reduction in negative emotions after visiting the website.

Among the participants with high suicidal thoughts at baseline, 54.1% (n=759) improved after viewing the website, with the top 3 reasons being “It distracted me” (n=190, 25%), “I felt less alone” (n=128, 16.9%), and “I learned something” (n=110, 14.5%). Among the participants with moderate suicidal thoughts at baseline, 49.8% (n=452) improved after viewing the website, with the top 3 reasons being “I felt less alone” (n=105, 23.2%), “I learned something” (n=100, 22.1%), and “It distracted me” (n=96, 21.2%). Among the participants with no suicidal thoughts at baseline, 39.8% (n=349) reported that they still “learned something,” and 14.7% (n=129) reported that it was helpful in “other” ways.

**Table 2:** The frequencies of reasons endorsed for why NowmattersNow.org was helpful overall and categorized by the intensity of BL^a^ suicidal thoughts and the change in suicidal thoughts (n=3185).

	No BL suicidal thoughts (1), n (%)				Moderate BL suicidal thoughts (2-3), n (%)					High BL suicidal thoughts (4-5), n (%)					Overall, n (%)				
	No change (n=857)	Worsened (n=19)	Overall (n=876)	Improved (n=452)		No Change (n=401)	Worsened (n=54)	Overall (n=907)	Improved (n=759)		No Change (n=624)	Worsened (n=19)	Overall (n= 1402)	Improved (n=1211)		No Change (n=1882)	Worsened (n=92)	Overall (n= 3185)	
I learned something	347 (40.5)^b^	2 (10.5)^c^	349 (39.8)^b^	100 (22.1)^d^		60 (15)^d^	7 (13)^c^	167 (20.1)^d^	110 (14.5)^c^		41 (6.6)	1 (5.3)	152 (10.8)	210 (17.3)^c^		448 (23.8)^b^	10 (10.9)^c^	668 (21)^b^	
It distracted me	86 (10)	6 (31.6)^b^	92 (10.5)	96 (21.2)^c^		79 (19.7)^b^	4 (7.4)	179 (21.6)^b^	190 (25)^b^		81 (13)^c^	2 (10.5)^c^	273 (19.5)^b^	286 (23.6)^b^		246 (13.1)^c^	12 (13)^d^	544 (17.1)^d^	
I felt less alone	78 (9.1)	1 (5.3)	79 (9)	105 (23.2)^b^		47 (11.7)^c^	9 (16.7)^d^	161 (19.4)^c^	128 (16.9)^d^		46 (7.4)	0 (0)	174 (12.4)^c^	233 (19.2)^d^		171 (9.1)	10 (10.9)^c^	414 (13)^c^	
Time passed	30 (3.5)	3 (15.8)^d^	33 (3.8)	34 (7.5)		44 (11)	4 (7.4)	51 (6.2)	73 (9.6)		59 (9.5)	5 (26.3)^b^	136 (9.7)	107 (8.8)		133 (7.1)	12 (13)^d^	252 (7.9)	
Other	129 (15.1)^d^	0 (0)	129 (14.7)^d^	13 (2.9)		29 (7.2)	2 (3.7)	31 (3.7)	23 (3)		42 (6.7)	0 (0)	65 (4.6)	36 (3)		200 (10.6)	2 (2.2)	238 (7.5)	
It wasn’t helpful	23 (2.7)	0 (0)	23 (2.6)	4 (0.9)		32 (8)	12 (22.2)^b^	48 (5.8)	7 (0.9)		136 (21.8)^d^	4 (21.1)^d^	147 (10.5)	11 (0.9)		191 (10.1)	16 (17.4)^b^	218 (6.8)	
I called/ texted a crisis line	7 (0.8)	2 (10.5)^d^	9 (1)	21 (4.6)		20 (5)	4 (7.4)	26 (3.1)	84 (11.1)		58 (9.3)	1 (5.3)	143 (10.2)	105 (8.7)		85 (4.5)	7 (7.6)	197 (6.2)	
I felt cared for	22 (2.6)	1 (5.3)	23 (2.6)	36 (8)		13 (3.2)	1 (1.9)	38 (4.6)	40 (5.3)		12 (1.9)	2 (10.5)^c^	54 (3.9)	76 (6.3)		47 (2.5)	4 (4.3)	127 (4)	
I saw that others could recover	36 (4.2)	1 (5.3)	37 (4.2)	14 (3.1)		17 (4.2)	2 (3.7)	20 (2.4)	36 (4.7)		9 (1.4)	0 (0)	45 (3.2)	50 (4.1)		62 (3.3)	3 (3.3)	115 (3.6)	
No response	99 (11.6)^c^	3 (15.8)^d^	102 (11.6)^c^	29 (6.4)		60 (15)^d^	9 (16.7)^d^	108 (13)	68 (9)		140 (22.4)^b^	4 (21.1)^d^	212 (15.1)^d^	97 (8)		299 (15.9)^d^	16 (17.4)^b^	412 (12.9)	

^a^BL: baseline.

^b^Most frequently endorsed reason.

^c^3rd most frequently endorsed reason.

^d^2nd most frequently endorsed reason.

**Table 3:** The frequencies of reasons endorsed for why NowmattersNow.org was helpful overall and categorized by the intensity of BL^a^ negative emotions and by the change in negative emotion (n=3185).

	No BL negative emotion (1), n (%)			Moderate BL negative emotion (2-3), n (%)				High BL negative emotion (4-5), n (%)					Overall, n (%)				
	No change (n=400)	Worsened (n=40)	Overall (n=440)	Improved (n=347)	No change (n=300)	Worsened (n=86)	Overall (N=733)	Improved (n= 1180)	No change (n=795)	Worsened (n=37)	Overall (N= 2012)	Improved (n=1527)		No change (n=1495)	Worsened (n=163)	Overall (n=3185)	
I learned something	164 (41)^b^	10 (25)^b^	174 (39.5)^b^	119 (34.3)^b^	83 (27.7)^b^	20 (23.3)^b^	222 (30.3)^b^	198 (16.8)^c^	69 (8.7)	5 (13.5)^c^	272 (13.5)^c^	317 (20.8)^d^		316 (21.1)^b^	35 (21.5)^b^	668 (21.1)^b^	
It distracted me	7 (17.5)	7 (17.5)^d^	14 (3.2)	45 (13)^d^	53 (17.7)^d^	9 (10.5)	107 (14.6)^d^	277 (23.5)^b^	115 (14.5)^c^	2 (5.4)	394 (19.6)^b^	322 (21.1)^b^		204 (13.6)^c^	18 (11)	544 (17.1)^d^	
I felt less alone	25 (6.3)	4 (10)	29 (6.6)	65 (18.7)^d^	29 (9.7)	9 (10.5)	103 (14.1)^c^	227 (19.2)^d^	52 (6.5)	3 (8.1)	282 (14)	292 (19.1)^c^		106 (7.1)	16 (9.8)	414 (13)^c^	
Time passed	6 (1.5)	3 (7.5)	9 (2)	14 (4)	30 (10)^c^	12 (14)^c^	56 (7.6)	102 (8.6)	81 (10.2)	4 (10.8)	187 (9.3)	116 (7.6)		117 (7.8)	19 (11.7)	252 (7.9)	
Other	78 (19.5)^d^	2 (5)	80 (18.2)^d^	25 (7.2)	29 (9.7)	5 (5.8)	59 (8)	36 (3.1)	62 (7.8)	1 (2.7)	99 (4.9)	61 (4)		169 (11.3)	8 (4.9)	238 (7.5)	
It wasn’t helpful	7 (1.8)	3 (7.5)	10 (2.3)	3 (0.9)	12 (4)	9 (10.5)	24 (3.3)	16 (1.4)	161 (20.3)^d^	7 (18.9)^d^	184 (9.1)	19 (1.2)		180 (12)	19 (11.7)^c^	218 (6.8)	
I called/ texted a crisis line	2 (0.5)	3 (7.5)	5 (1.2)	10 (2.9)	7 (2.3)	3 (3.5)	20 (2.7)	99 (8.4)	68 (8.6)	5 (13.5)^c^	172 (8.5)	109 (7.1)		77 (5.2)	11 (6.7)	197 (6.2)	
I felt cared for	9 (2.3)	0 (0)	9 (2.2)	26 (7.5)	12 (4)	0 (0)	38 (5.2)	69 (5.8)	11 (1.4)	0 (0)	80 (4)	95 (6.2)		32 (2.1)	0 (0)	127 (4)	
I saw that others could recover	15 (3.8)	3 (7.5)	18 (4.4)	14 (4)	12 (4)	6 (7)	32 (4.4)	50 (4.2)	13 (1.6)	2 (5.4)	65 (3.2)	64 (4.2)		40 (2.7)	11 (6.7)	115 (3.6)	
No response	58 (14.5)^c^	5 (12.5)^c^	63 (15.3)^c^	26 (7.5)	33 (11)	13 (15.1)^d^	72 (9.8)	106 (9)	163 (20.5)^b^	8 (21.6)^b^	277 (13.8)^d^	132 (8.6)		254 (17)^d^	26 (16)^d^	412 (12.9)	

^a^BL: baseline.

^b^Most frequently endorsed reason.

^c^3rd most frequently endorsed reason.

^d^2nd most frequently endorsed reason.

Among the participants with high negative emotions at baseline, 58.6% (n=1180) improved after viewing the website, with the top 3 reasons being “It distracted me” (n=277, 23.5%), “I felt less alone” (n=227, 19.2%), and “I learned something” (n=198, 16.8%). Among the participants with moderate negative emotions at baseline, 47.3% (n=347) improved after viewing the website, with the top 3 reasons being “I learned something” (n=119, 34.3%), “I felt less alone” (n=65, 18.7%), and “It distracted me” (n=45, 13%). Among the participants with no negative emotions at baseline, 39.5% (n=174) reported that they still “learned something,” and 18.2% (n=80) reported that it was helpful in “other” ways.

Tables 4 and 5 summarize participants’ reasons for finding NowMattersNow.org helpful, categorized by the magnitude of change in their suicidal ideation and negative emotions. For participants who experienced the largest reduction in suicidal ideation (ie, a 4-point decrease), the most common reasons cited were “It distracted me” (n=5, 29.4%), “I felt less alone” (n=3, 17.6%), and “I felt cared for” (n=3, 17.6%), with 17.6% (n=3) of participants not responding. Among those who showed smaller reductions in suicidal ideation (ie, a 1-, 2-, or 3-point decrease), the top reasons were similar: “It distracted me,” “I felt less alone,” and “I learned something.” For the few participants who reported the greatest increase in suicidal ideation (ie, a 4-point increase), one indicated they “called/texted a crisis line,” while the other reported that “time passed.” Participants with smaller increases in suicidal ideation (ie, a 1-, 2-, or 3-point increase) provided varied responses, which are detailed in Table 4. Among participants who reported no change in the magnitude of suicidal ideation (ie, a 0-point change), 23.8% (n=448) cited “I learned something” as helpful, 15.9% (n=299) did not respond, and 13.1% (n=246) noted “It distracted me.”

Similarly, for those who experienced the largest reduction in negative emotions (ie, a 4-point decrease), 23.1% (n=3) reported “It distracted me,” 23.1% (n=3) “I felt less alone,” and 15.4% (n=2) “I felt cared for,” with 21.3% (n=2) not responding. Participants who reported smaller reductions in negative emotions (ie, a 1-, 2-, or 3-point decrease) cited similar reasons, including “It distracted me,” “I felt less alone,” and “I learned something.” Among participants with the largest increase in negative emotions (ie, a 4-point increase), 2 reported they “called/texted a crisis line,” 2 indicated “time passed,” 1 mentioned “It distracted me,” and 1 stated, “It wasn’t helpful.” For those with smaller increases (ie, a 1-, 2-, or 3-point increase), responses were varied and are detailed in Table 5. For participants with no change in negative emotions (ie, a 0-point change), 21.1% (n=316) noted “I learned something,” 17% (n=254) did not respond, and 13.6% (n=204) mentioned “It distracted me.”

**Table 4:** The frequencies of reasons endorsed for why NowMattersNow.org was helpful, categorized by the magnitude of change in suicidal thoughts (n=3185).

	Change in suicidal thoughts, n (%)									
	4-point decrease (–4)	3-point decrease (–3)	2-point decrease (–2)	1-point decrease (–1)	No Change (0)	1-point increase (+1)	2-point increase (+2)	3-point increase (+3)	4-point increase (+4)	
It distracted me	5 (29.4)	13 (14.8)	81 (23.5)	187 (24.5)	246 (13.1)	9 (13)	3 (16.7)	0 (0)	0 (0)	
I felt less alone	3 (17.6)	19 (21.6)	63 (18.3)	148 (19.4)	171 (9.1)	7 (10.1)	3 (16.7)	0 (0)	0 (0)	
I learned something	1 (5.9)	10 (11.4)	64 (18.6)	135 (17.7)	448 (23.8)	7 (10.1)	1 (5.6)	2 (66.7)	0 (0)	
I called/texted a crisis line	2 (11.8)	6 (6.8)	38 (11)	59 (7.7)	85 (4.5)	3 (4.3)	2 (11.1)	1 (33.3)	1 (50)	
I saw that others could recover	0 (0)	12 (13.6)	12 (3.5)	26 (3.4)	62 (3.3)	3 (4.3)	0 (0)	0 (0)	0 (0)	
I felt cared for	3 (17.6)	10 (11.4)	22 (6.4)	41 (5.4)	47 (2.5)	4 (5.8)	0 (0)	0 (0)	0 (0)	
Time passed	0 (0)	5 (5.7)	30 (8.7)	72 (9.4)	133 (7.1)	9 (13)	2 (11.1)	0 (0)	1 (50)	
Other	0 (0)	1 (1.1)	7 (2)	28 (3.7)	200 (10.6)	0 (0)	2 (11.1)	0 (0)	0 (0)	
It wasn’t helpful	0 (0)	0 (0)	2 (0.6)	9 (1.2)	191 (10.1)	15 (21.7)	1 (5.6)	0 (0)	0 (0)	
No response	3 (17.6)	12 (13.6)	25 (7.3)	57 (7.5)	299 (15.9)	12 (17.4)	4 (22.2)	0 (0)	0 (0)	

**Table 5:** The frequencies of reasons endorsed for why NowMattersNow.org was helpful, categorized by the magnitude of change in negative emotions (n=3185).

	Change in negative emotions, n (%)								
	4-point decrease (–4)	3-point decrease (–3)	2-point decrease (–2)	1-point decrease (–1)	No Change (0)	1-point increase (+1)	2-point increase (+2)	3-point increase (+3)	4-point increase (+4)
It distracted me	3 (23.1)	17 (18.9)	90 (19.5)	212 (22)	204 (13.6)	11 (9.2)	5 (14.7)	1 (25)	1 (16.7)
I felt less alone	3 (23.1)	14 (15.6)	84 (18.2)	191 (19.8)	106 (7.1)	11 (9.2)	4 (11.8)	1 (25)	0 (0)
I learned something	1 (7.7)	16 (17.8)	92 (20)	208 (21.6)	316 (21.1)	29 (24.4)	5 (14.7)	1 (25)	0 (0)
I called/texted a crisis line	1 (7.7)	6 (6.7)	40 (8.7)	62 (6.4)	77 (5.2)	8 (6.7)	1 (2.9)	0 (0)	2 (33.3)
I saw that others could recover	0 (0)	5 (5.6)	29 (6.3)	30 (3.1)	40 (2.7)	6 (5)	5 (14.7)	0 (0)	0 (0)
I felt cared for	2 (15.4)	9 (10)	37 (8)	47 (4.9)	32 (2.1)	0 (0)	0 (0)	0 (0)	0 (0)
Time passed	0 (0)	5 (5.6)	35 (7.6)	76 (7.9)	117 (7.8)	16 (13.4)	1 (2.9)	0 (0)	2 (33.3)
Other	0 (0)	0 (0)	12 (2.6)	49 (5.1)	169 (11.3)	4 (3.4)	4 (11.8)	0 (0)	0 (0)
It wasn’t helpful	0 (0)	0 (0)	3 (0.7)	16 (1.7)	180 (12)	13 (10.9)	4 (11.8)	1 (25)	1 (16.7)
No response	3 (23.1)	18 (20)	39 (8.5)	72 (7.5)	254 (17)	21 (17.6)	5 (14.7)	0 (0)	0 (0)

## Discussion

### Principal Findings

This study builds upon our previous research supporting NowMattersNow.org for the temporary relief of suicidal ideation and negative emotions [16]. Here, we present findings from a concise user-experience questionnaire completed by visitors to NowMattersNow.org. In addition to assessing changes in suicidal thoughts and negative emotions during website use, the questionnaire also explored users’ perceptions of the site’s helpfulness. This paper aimed to examine the reasons behind reductions in suicidal thoughts and negative emotions reported after using the site. Our hypothesis—that participants who experienced reductions in suicidal thoughts and negative emotions would attribute this improvement primarily to “feeling less alone”—was mostly supported. Over half of the participants who reported suicidal ideation (n=1211, 52.4%) or negative emotions (n=1527, 55.6%) upon arriving at the site experienced reductions in these symptoms after engaging with NowMattersNow.org. Specifically, over half of those with high baseline suicidal thoughts experienced improvement, citing “It distracted me,” “I felt less alone,” and “I learned something” as the top reasons for the website’s helpfulness. Similarly, almost half of the participants with moderate baseline suicidal thoughts also showed improvement; for them, “I felt less alone” was the most frequently endorsed reason, followed by “I learned something” and “It distracted me.” While feeling less alone was not the most common reason cited by those with the highest levels of suicidal ideation, it was the second most helpful factor and ranked as the most helpful for those with moderate suicidal ideation. These findings align with leading theories of suicide [33,34], which propose that social disconnection is a key driver of suicidal ideation. Thus, it is expected that individuals who feel less alone after visiting NowMattersNow.org would experience a decrease in the intensity of their suicidal thoughts. The site’s focus on sharing others’ recovery stories likely fosters a sense of community and connection, allowing visitors to feel understood and less isolated. This sense of shared experience may be a core “active ingredient” of NowMattersNow.org, suggesting that digital mental health tools aimed at suicide prevention might benefit from emphasizing social connectedness and community support.

For reductions in negative emotions, more than half of individuals with high baseline negative emotions reported improvement, with “It distracted me,” “I felt less alone,” and “I learned something” as the main reasons they found the website helpful. Similarly, nearly half of individuals with moderate baseline negative emotions improved, citing “I learned something,” “I felt less alone,” and “It distracted me” as the top reasons for the website’s helpfulness. These patterns mirror those observed among participants with high and moderate levels of suicidal ideation, supporting the idea that fostering social connectedness can reduce not only suicidal thoughts but also negative emotions more broadly. An important point is that distraction during moments of crisis—when experiencing intense suicidal ideation or negative emotions—is a key skill taught in DBT to help patients manage acute distress [25]. The NowMattersNow.org website appears well-suited to fulfill this function for its users, providing an immediate coping tool. Additionally, participants with high or moderate levels of suicidal ideation or negative emotions who reported improvement also listed “I learned something” among their top reasons for the site’s helpfulness. This suggests that NowMattersNow.org is not only a valuable support tool but also an educational resource that may empower individuals with knowledge that alleviates distress. These findings highlight 3 potential active ingredients for reducing suicidal ideation and negative emotions: promoting social connectedness, offering distraction, and providing educational content. By incorporating these elements, NowMattersNow.org may be effectively addressing the core needs for individuals in distress.

The observed reductions in suicidal ideation (0.5-point reduction) and negative emotions (0.62-point reduction) in this study correspond to percentage decreases of 12.5% and 15.5%, respectively. While these changes are statistically significant, they are relatively modest, and it remains an open empirical question whether such reductions are clinically meaningful. However, the authors believe that even these modest reductions could be clinically significant, especially considering that they were achieved after only one session. Although we cannot precisely determine how much time participants spent on the website, it is reasonable to assume that most engaged with it in a single sitting. A 12.5% reduction in suicidal thoughts and a 15.5% reduction in negative emotions could represent a shift from a crisis mode to a state where individuals can more readily use coping skills or seek additional help, potentially preventing maladaptive behaviors. For context, research on session-to-session anxiety and depression symptom reductions in therapies like CBT reports decreases ranging from 4% to 8% [40,41]. Therefore, a 12.5% reduction in suicidal ideation following just one visit to the website could have meaningful implications for the user’s emotional state and overall well-being.

This study also explored which reported reasons were most strongly associated with the largest improvements in suicidal ideation and negative emotions. Given the limited research on the specific mechanisms through which websites exert their effects, we did not have a prespecified hypothesis about which factors would be linked to different levels of improvement. Participants who reported the largest reductions in suicidal ideation cited “It distracted me,” “I felt less alone,” and “I felt cared for” as the top reasons the website was helpful. Those with moderate improvements in suicidal ideation cited similar reasons, including distraction, feeling less alone, and learning something. The top 3 reasons for reductions in negative emotions closely mirrored those for suicidal ideation, with distraction, social connection, and learning cited as key contributors to improvements. These findings suggest that fostering social connection, offering distraction, and providing educational content may not only reduce suicidal ideation and negative emotions overall but may also lead to the most significant improvements. Prioritizing these elements in digital mental health resources like NowMattersNow.org could, therefore, maximize their impact and effectiveness for individuals in distress.

This study also gathered demographic information on vulnerable populations, including LGBTQI individuals, American Indian or Alaska Native individuals, those with alcohol or substance use issues, and those reporting unusual experiences. While participants in the full sample showed an average reduction of half a point in suicidal ideation intensity and more than half a point in negative emotions, certain groups experienced even greater benefits. LGBTQI-identifying individuals reported more significant reductions in both suicidal ideation and negative emotions compared to non-LGBTQI individuals. Similarly, those with alcohol or opioid use issues showed greater reductions in suicidal ideation than those without these issues. Additionally, individuals who reported unusual experiences experienced greater decreases in both suicidal ideation and negative emotions than those who did not report such experiences. The reasons participants provided for these reductions were consistent with those reported across the full sample, often reflecting themes such as distraction (“It distracted me”), connection (“I felt less alone”), and learning (“I learned something”). These findings highlight NowMattersNow.org’s potential to effectively support vulnerable groups by addressing unique challenges that conventional mental health resources may overlook. The site’s content appears to resonate particularly well with individuals who identify as LGBTQI, have substance use issues, or experience unusual mental health symptoms, likely because it fosters connection, validation, and immediate coping strategies that reduce distress. This suggests that digital interventions tailored to the needs of high-risk populations can serve as accessible, impactful resources, offering a crucial support option for those who might otherwise remain isolated in their distress.

In summary, this study sheds light on the mechanisms behind the effectiveness of NowMatttersNow.org in reducing suicidal ideation and negative emotions, with 80% (n=2555) of respondents indicating that the website was helpful. Key features identified by visitors included promoting social connectedness, offering distraction, and providing educational content. The inclusion of personal stories from individuals with lived experience may improve users’ sense of connection and support. This finding aligns with research suggesting that adolescents, in particular, benefit from suicide prevention narratives that feature peer experiences and coping strategies [42]. Future studies should aim to collect other demographic data, such as age, to determine how it moderates the perceived helpfulness of specific website features. This study highlights the potential for NowMattersNow.org to serve as a scalable, accessible, and immediate resource for reducing suicidal ideation and negative emotions, especially for those who face barriers to traditional mental health care, including individuals from vulnerable populations. While challenges such as technical issues [43] and the complexity of web-based interactions [44] exist—often due to many websites not being designed with users in mind [45]—digital websites like NowMattersNow.org should not be viewed as replacements for in-person care. Instead, they may help bridge the gap when face-to-face support is unavailable or augment ongoing in-person treatment. Providers recommending these resources should avoid offering them as standalone interventions and ensure they are paired with psychotherapy whenever possible. However, using these tools as interim support for patients on waitlists is appropriate. While reliance on web-based tools as the sole form of treatment is discouraged, individuals should not be deterred from exploring them independently when other care options are inaccessible or undesired. Continued research is important to better understand the needs of individuals seeking digital mental health support, as well as to improve user-friendliness, ensuring that these resources are comprehensible and accessible to individuals from all backgrounds.

### Strengths and Limitations

These findings should be interpreted within the context of the study’s strengths and limitations. A key strength of this study was the large sample size (n=3185), which afforded higher statistical power to evaluate overall and subgroup differences in suicidal ideation and negative emotions. A related limitation of large sample sizes is the need to temper the interpretation of the observed reductions in suicidal ideation and negative emotions, as small effects are more likely to reach statistical significance with a large sample size. Specifically, the average reductions in suicidal ideation and negative emotions were approximately a half-point on the 5-point scales, which were relatively modest. These analyses adjusted for a limited number of characteristics associated with suicide risk (eg, alcohol or opioid misuse), but we were unable to adjust for other relevant risk factors (eg, age).

This study used a single-arm pragmatic trial design, using quality improvement data from the NowMattersNow.org web-based platform, with permission to use the data for research purposes. By adopting a real-world approach, this study allowed for natural variations in how users interacted with the site, rather than imposing rigid use protocols. This is in contrast to traditional experimental designs, where standardized engagement and control groups are the norm. As a result of its pragmatic nature, certain elements commonly found in randomized controlled trials were not present in this study. For instance, there was no pre- or postintervention assessment using validated questionnaires, and the outcomes were evaluated retrospectively. Additionally, due to the real-world setting of the trial, no control group was included for comparison. These factors, while limiting the ability to draw definitive causal conclusions, are in line with the pragmatic design’s emphasis on ecological validity and generalizability. The strength of this pragmatic trial lies in its real-world context: it was conducted with actual users of the NowMattersNow.org website, who interacted with the platform in their own way, reflecting typical use patterns. This design closely mirrors how digital interventions are used in practice, providing valuable insights into the website’s potential impact on those at risk of suicide, as it would occur in everyday settings outside the constraints of a clinical trial. The findings, therefore, have strong implications for understanding how such interventions can be applied in the broader community, offering actionable insights for optimizing digital suicide prevention resources.

A significant limitation is that user-experience data were gathered from individual users at a single point in time, and the lack of a control group for comparison. As such, it is possible that the observed improvements were influenced by distraction alone or merely the passage of time, which could lead to similar reductions in suicidal ideation and negative emotions. Additionally, we cannot determine whether the reported improvements were sustained beyond the brief timeframe covered by the survey. Nonresponse bias is another concern that may affect the generalizability of these findings. Individuals who viewed the survey but chose not to complete it, as well as those who never encountered the survey, may differ in their experiences and responses, particularly if they did not have positive experiences with the website. Another limitation of the study is the use of the term “unusual experiences,” which was not explicitly defined for participants. As a result, the interpretation of this term was left to individual interpretation, and it is unclear how participants construed this option. As such, the exact nature of “unusual experiences” within the context of this study remains unknown. Future studies should provide a clearer definition of this term and explore participants’ interpretations to gain a more precise understanding of the experiences being referred to. Further, limitations related to IP address tracking should be acknowledged. To avoid duplicate responses, records with the same IP snippet were removed, which means that participants sharing a communal network (eg, within the same facility) may have been unintentionally excluded. Conversely, individuals who accessed the website from different locations may have been assigned different IP addresses, potentially leading to multiple records from the same participant. These issues highlight challenges in accurate participant counts and representative sampling in web-based research.

The sensitive nature of the survey topic (suicide) may have influenced participation; some individuals, particularly those experiencing self-stigma or discrimination, might have been less likely to participate, while others who found the topic highly relevant may have been more inclined to engage, including those at a higher risk for suicide. Furthermore, participants rated their suicidal thoughts and negative emotions before and after using the website within a single survey. While this approach was intended to encourage participation, it raises the possibility of recall bias, which could impact the accuracy of reported reductions in distress. Although the survey demonstrates face validity, other forms of validity have not been assessed, and it remains unclear whether the respondents accurately reflect other individuals experiencing suicidal thoughts. Future randomized controlled trials are crucial to determine the causal relationships between website use and its features and reported improvements. Additionally, longitudinal studies are necessary to assess the long-term impact of the website on users’ mental health, allowing for insights into the sustainability of any observed benefits. Finally, researchers should investigate whether personal narratives do, in fact, lead to improved social connectedness.

### Conclusions

This study improves our understanding of the mechanisms by which NowMattersNow.org provides relief from suicidal ideation and negative emotions. With 80% (n=2555) of respondents finding the website helpful, the research identified critical features that contribute to its effectiveness, namely, through social connectedness, distraction, and educational content. The inclusion of personal narratives from individuals with lived experience appears to resonate deeply with users, fostering a sense of connection that aligns with existing literature emphasizing the importance of social support in suicide prevention. Furthermore, our findings suggest that NowMattersNow.org may be particularly helpful for vulnerable populations, such as LGBTQI individuals and those with substance use issues, highlighting the platform’s role in addressing unique challenges often overlooked by conventional mental health resources. It is crucial that this resource not be introduced via asynchronous messaging from an unknown provider. When offered by a current care provider, we recommend it be reviewed together during the initial encounter and revisited in follow-up sessions to avoid a delivery style associated with increased self-harm in a previous pragmatic clinical trial [31]. As digital interventions like NowMattersNow.org continue to evolve, it is vital to conduct further research that includes diverse demographic data to optimize their design and functionality. By prioritizing user needs and improving accessibility, NowMattersNow.org can serve as an immediate resource for those seeking self-help, including those who may encounter challenges in accessing conventional mental health services, offering crucial resources when direct, in-person options are not feasible. NowMattersNow.org can be a resource for individuals facing barriers to traditional mental health care, potentially bridging the gap when in-person support is unavailable and ensuring that individuals from all backgrounds can find meaningful support.

## Abbreviations

CBT: cognitive behavioral therapy
DBT: dialectical behavior therapy
STB: suicidal thought and behavior

## Data Availability

The datasets generated and analyzed in this study are not publicly available, as participants did not provide consent for their data to be shared beyond quality improvement efforts and research conducted by NowMattersNow.org, but are available from the corresponding author on reasonable request.
